# African-Caribbean cancer consortium for the study of viral, genetic and environmental cancer risk factors

**DOI:** 10.1186/1750-9378-2-17

**Published:** 2007-09-24

**Authors:** Camille C Ragin, Emanuela Taioli, Norma McFarlane-Anderson, Gordon Avery, Franklyn Bennett, Adelia Bovell-Benjamin, Angela Brown Thompson, Agatha Carrington, Lydia Campbell-Everett, Jacqueline Ford, Anselm Hennis, Maria Jackson, Sandra Lake, M Cristina Leske, Carol Magai, Barbara Nemesure, Alfred Neugut, Folakemi Odedina, Michael Okobia, Alan Patrick, Wallis Best Plummer, R Renee Reams, Robin Roberts, Sharaneen Scott-Hastings, Sangita Sharma, Victor Wheeler, Suh-Yuh Wu, Clareann Bunker

**Affiliations:** 1 Department of Epidemiology, University of Pittsburgh Graduate School of Public Health, Pittsburgh, PA, USA.; 2 The University of Pittsburgh Cancer Institute, Pittsburgh, PA, USA.; 3 Faculty of Medical Sciences, University of the West Indies, Kingston, Jamaica.; 4 Leeward Islands Health Research Unit, Medical University of the Americas, Charlestown, Nevis, USA.; 5 Department of Pathology, University of the West Indies, Kingston, Jamaica.; 6 Department of Food and Nutritional Sciences, Tuskegee University, Tuskegee, AL, USA.; 7 Department of Health, St. James, Jamaica.; 8 Northwest Regional Health Authority, Ministry of Health, Trinidad and Tobago.; 9 Triune Ob/Gyn Services, LLC, Brooklyn, NY, USA.; 10 Chronic Disease Research Centre, Univ. of the West Indies, Bridgetown, Barbados.; 11 Myeloma Lymphoma & Leukemia Foundation of Barbados, Barbados.; 12 Department of Preventive Medicine, Stony Brook University, Stony Brook, NY, USA.; 13 Department of Psychology, Long Island University, Brooklyn, NY, USA.; 14 Columbia University, Herbert Irving Comprehensive Cancer Center, New York, NY, USA.; 15 College of Pharmacy & Pharmaceutical Sciences, Florida A&M University, Tallahassee, FL, USA.; 16 The Tobago Health Studies Office, Scarborough, Trinidad and Tobago.; 17 Department of Pharmacy, University of Guyana, Turkeyen, Guyana.; 18 Hospital Authority, Nassau, Bahamas.; 19 Cancer Etiology Program, Cancer Research Center of Hawaii, University of Hawaii, Honolulu, HI, USA.

## Abstract

This is a short summary of a meeting of the "African-Caribbean Cancer Consortium", jointly organized by the University of Pittsburgh, Department of Epidemiology and the University of Pittsburgh Cancer Institute, held in Montego Bay, Jamaica as a satellite meeting at the Caribbean Health Research Council, 52^nd ^Annual Council and Scientific meeting on May 4, 2007.

## Introduction

The University of Pittsburgh Graduate School of Public Health and the University of Pittsburgh Cancer Institute organized in Montego Bay, Jamaica a satellite meeting at the 52^nd ^Annual Council and Scientific meeting of the Caribbean Health Research Council, on May 4, 2007, for the purpose of introducing the concept of an African-Caribbean Cancer Consortium (AC^3^) to research investigators from the Caribbean islands, and to extend an invitation to join the Cancer Consortium.

There were 18 attendees from various countries: Bahamas, Barbados, Guyana, Hawaii, Jamaica, St. Kitts/Nevis, Trinidad and Tobago, and the United States; the majority of whom presented or discussed their work and research interests. All of them expressed intent to participate as investigators in the proposed Cancer Consortium.

Participants agreed that the initial purpose of the AC^3 ^will be the study of viral, genetic, environmental, and lifestyle/behavioral risk factors for cancer in populations of African descent. The primary goal will be to provide new collaborative opportunities for cancer research between the United States, Africa, and the Caribbean. The AC^3 ^aims to 1) address a significant need for studies related to cancer for individuals of African descent 2) advance scientific knowledge of the roles that viral, environmental, and genetic risk factors play in cancer etiology among minority populations and 3) lead to targeted interventions in order to address the existing disparity by reducing the incidence and mortality rates of cancer in these minority populations.

## Existing collaborations

Several collaborations are already ongoing between US, Caribbean and African  investigators. These include studies of prostate, breast, and cervical cancers conducted in Africa, the Caribbean, as well as immigrant populations in the United States (Table 1).

**Table 1:** Existing collaborations involving U.S., Caribbean and African Investigators

**Project Location**	**Study**	**Investigators**
**University of Pittsburgh, Graduate School of Public Health**		
Nigeria	Breast cancer	Dr. Michael Okobia Dr. Clareann Bunker
Jamaica	HPV – cervical cancer	Dr. Norma McFarlane-Anderson Dr. Camille Ragin
Tobago	HPV – cervical cancer	Dr. Camille Ragin Dr. Clareann Bunker Dr. Alan Patrick Dr. Victor Wheeler
Trinidad & Tobago	HHV8 – prostate cancer	Dr. Clareann Bunker Dr. Alan Patrick Dr. Victor Wheeler
		
**Columbia and Long Island Universities**		
New York	Cancer in immigrant	Dr. Alfred Neugut
	Caribbean populations	Dr. Carol Magai
		
**State University of New York (Stony Brook)**		
Barbados	Barbados National Cancer Study (Prostate and Breast cancers)	Dr. M. Cristina Leske Dr. Anselm Hennis Dr. Barbara Nemesure Dr. Suh-Yuh Wu
		
**Florida A & M University**		
USA	Prostate cancer	Dr. R. Renee Reams
Nigeria & USA	Prostate cancer	Dr. Folakemi Odedina

The work by Phillips et al. [1] was reviewed to discuss the higher rates of cancers in the cervix, esophagus, liver, and stomach in the Caribbean islands in comparison with the United States. All of these cancers have been demonstrated to be etiologically linked to, or associated, with infectious agents. Cervical and liver cancers are associated with Human Papillomavirus (HPV) and Hepatitis B & C viruses respectively, while researchers are exploring the possible associations of HPV with esophageal cancer and Helicobacter pylori with stomach cancers [2-5]. Other viral-cancer relationships that might be of interest to Caribbean populations are Human Herpesvirus 8 (HHV8) and prostate cancers, as well as Human T leukemia virus – 1 (HTLV1) and Adult T-cell Leukemia/lymphoma.

A review of the literature on the prevalence of cancer-associated viral infections in healthy Caribbean populations was presented [6] and compared to the prevalence of each of these viral infections in the US. For all the viruses studied, with the exception of HCV, the Caribbean had significantly higher prevalence rates than the US. For the Caribbean, the incidence of cervical cancer ranks third among all cancers in females (Age-standardized rate (ASR) = 32.62/100,000), while liver cancer ranks sixth among Caribbean females (ASR = 4.54/100,000) and fifth among Caribbean males (ASR = 8.16/100,000) [7]. In contrast, cervical cancer among US females is ranked 17^th ^for all cancers (ASR = 7.65/100,000) and for US males, liver cancer is ranked 15^th ^for all cancers (ASR = 5.47/100,000) [7]. Therefore, at least for HPV and HBV infections, the elevated rates in the Caribbean appeared to fit well with the known epidemiological information on incidence of these cancers in the Caribbean.

Several studies have been conducted on cervical dysplasia, cancer, and HPV infection in Jamaican women (Dr. Norma McFarlane-Anderson, University of the West Indies, Mona Campus). This work focused on investigations of lifestyle and genetic susceptibility factors related to cervical dysplasia and cancer, as well as HPV prevalence and genotype distribution among healthy Jamaican women. The report demonstrated a high frequency of HPV infection [8], which corresponds with the high rate of cervical cancer in Jamaica (27.6/100,000) [9]. An HPV vaccine for prevention of cervical cancer has been recently approved by the Federal Drug Administration (FDA) and targets the most common high-risk HPV types, 16 and 18. It is expected that implementation of this vaccine would prevent approximately 70–75% of all cervical cancers worldwide. In Africa, Central and South America, and Asia, a larger proportion of cervical cancer cases are associated with HPV types other than 16 and 18 (41%–36%), compared to the cervical cancer cases among females from Europe, US, and Australia (~25%) [10]. Currently, there are no data that describes the proportion of HPV16 and HPV18-positive cervical cancer cases in the Caribbean islands. Therefore, there is a need for additional studies in this population, particularly now with the advent of the new HPV vaccine. Some of the data presented by Dr. Norma McFarlane-Anderson were generated through an existing collaboration with US investigators, further emphasizing the role that the AC^3 ^could play in providing these sorts of collaborative opportunities.

The work on prostate cancer risk in Tobago men was summarized (Dr. Clareann Bunker, University of Pittsburgh and Tobago Health Studies) [11-16]. A high rate of Human Herpesvirus 8 (HHV8) infection in Tobago population was observed. Dr. Clareann Bunker also reported that short Androgen Receptor alleles have been shown to be associated with efficient androgen metabolism. Prostate cancer cases in the Tobago population were almost three times more likely to be HHV8 positive and have short repeat Androgen Receptor alleles than controls of similar age. Additional studies to define the biological mechanisms which underlie this association are ongoing.

The Barbados National Cancer Study (BNCS) (Dr. Anselm Hennis) is a comprehensive epidemiological study of breast and prostate cancer to determine the incidence, environmental, and familial/genetic risk factors associated with these cancers. Some preliminary data on recruitment of breast and prostate cancer cases and controls into this study were presented. According to 2002 data from the International Agency for Cancer Research (IARC) [7], world-adjusted mortality rates*100,000 for prostate cancer are 16, 28 and 16 for W. Africa, the Caribbean, and the US, respectively, with incidences being 19, 52 and 125. Comparable data for breast cancer mortality is 20, 13 and 19, while incidence is 28, 33 and 101 *100,000. Dr. Anselm Hennis reported that Barbados' incidence and mortality rates of both cancers are the highest in the Caribbean, thus strengthening the importance of studying the African-Barbadian population.

## Existing and developing cancer registries

There are to date seven established cancer registries (Bahamas, Cuba, Guyana, Jamaica, Martinique, Trinidad and Tobago, and the Netherlands Antilles) as well as a prostate cancer registry in Grenada (Table 2).

**Table 2:** Existing cancer registries in the Caribbean

**Country**	**Type of Registry **	**Year of Establishment**	**Estimated Mid-Year Population (2007) [22]**
Cuba	National Cancer Registry	1964	11,416,987
Bahamas	National Cancer Registry	?	305,655
Grenada	Prostate Cancer Registry	1996	92,014
Guyana	National Cancer Registry	2000	769,095
Jamaica	Urban Cancer Registry^†^	1959	2,780,132
Martinique	National Cancer Registry	1983	439,202
Netherlands Antilles*	National Cancer Registry	1977	223,472
Trinidad and Tobago	National Cancer Registry	1994	1,056,608

*Includes six Dutch-Caribbean islands (Curacao, Aruba, Bonaire, Saba, St. Eustatius and St. Maarten)

^†^The Jamaican cancer registration currently occurs in urban areas only (Kingston and St. Andrew).

Cancer registration in Martinique has been mandatory since 1981 and the island's cancer registry was established in 1983 [17]. Cancer cases are identified through a number of mechanisms: medical records, data from private and public hospitals, laboratory records, as well as regional insurance records of the national public medical insurance system. For the period 1981–2000, there were 8,992 cancer cases in males and 6,832 cancer cases in females. The highest ranking cancer type in males from Martinique was prostate cancer (3,518 cases, world standardized incidence (WSI) = 80.83/100,000) followed by stomach cancer (849 cases, WSI = 20.86/100,000). Females had the highest incidence of breast cancer (1,568 cases, WSI = 35.80/100,000), followed by cervical cancer (890 cases, WSI = 20.82/100,000).

In Cuba, the National Cancer Registry was established in 1964 and documents cancer cases through the hospital system where the clinicians report the diagnosed cases, as well as through the examination of death certificates [18]. This cancer registry has provided a valuable tool to evaluate the burden of cancer in Cuba. The information from the cancer registry contributed to future developments of the country's National Cancer Control Program which commenced in 1989 [19].

The Netherlands Antilles, formerly a Dutch colony and now autonomous, consists of six small Caribbean islands (Curacao, Aruba, Bonaire, Saba, St. Eustatius and St. Maarten). A cancer registry was established in 1977 [20]. The establishment of a cancer registry in this region was accomplished through a unique combination of conditions that favored a highly reliable registration of cancer cases. There was a centralized Pathology Laboratory, direct access to laboratory and hospital records for critical evaluation of each individual case prior to registration. There was also cooperation of hospitals and physicians.

The Dr Elizabeth Quamina National Cancer Registry of Trinidad and Tobago (Ms Veronica Roach, Registrar) is a resource established in 1994 by the late Dr. Elizabeth Quamina. The registry includes data through passive and active collection from all sources where cancer is diagnosed, and has the capabilities to measure the burden of cancer in Trinidad and Tobago; determines incidence and mortality rates for all cancers; identifies cancer clusters and trends; informs government policies; satisfies requests for information from medical and non medical personnel; and disseminates information and analysis of data to health professionals and the general public through the publication of reports. The Trinidad and Tobago Registry presentation was done by Dr. Alan Patrick, on behalf of Ms. Veronica Roach. From this registry, it is apparent that the top two incident cancers in Trinidad and Tobago are prostate and colon/rectum cancers for males, and cervical and breast cancers for females. This cancer registry is capable of providing data to support cancer research priorities; however the requests for information have been infrequent.

The cancer registry of Guyana was established in 2000 [21] as an independent body, but is now a Department of the Ministry of Health (Dr. Wallis Plummer on behalf of Nurse Penelope Layne, Registrar). Significant effort has gone into enhancing the system of registration and reporting of cases, and both active and passive data collection is encouraged. The registry demonstrates both alarmingly high incidence and mortality rates of cervical, breast, and prostate cancers. Unfortunately, despite this important data, limited resources and a lack of expertise have prevented further work from being done to investigate the determinants of these rising cancer rates.

The Jamaican Cancer Registry was established in 1959 (Dr. Norma McFarlane-Anderson) and serves the eastern regions of Kingston and St. Andrew. There are discussions for the establishment of a cancer registry that will support the western region of the island. Barbados will soon have its own cancer registry (Dr. Anselm Hennis).

Dr. Robert Yearwood, the only urologist in Grenada, has established a prostate cancer registry since 1996 with 300 patients to date. There have been 28 new prostate cancer cases that have been registered since the beginning of this year (Dr. Robert Yearwood, personal communication). Based on a review of the pathology records, Dr. Robert Yearwood also reported that there have been 13 cases of breast cancer, and 10 cases of cervical and uterine cancers diagnosed this year.

Bahamas currently has a national cancer registry (Dr. Robin Roberts), and observations of high prostate cancer rates in this country were reported. The Bahamas National Cancer Registry is in its most embryonic stage, and documents only the cancers reported in the government's health care facilities and in the government's official health publications and reports. Very few health care providers are aware of its existence. Dr. Robin Roberts indicated that there is a great need to upgrade the cancer registry's function; expand its data base; and advance its utility, reliability, and relevance.

This information emphasizes the need for other Caribbean islands to have their own cancer registries. The participants of this meeting agreed that the development of cancer registries should be addressed at the next Consortium meeting. The Cancer Registry of Trinidad and Tobago should be used as a template in order to better understand the issues and requirements related to the establishment of a cancer registry, to standardize registries throughout the Caribbean, and to allow for pan-Caribbean comparisons to be made.

## Current limitations

Despite the amount of data generated by spontaneous studies in the Caribbean islands, several limitations were highlighted during this meeting. These included the lack of data on cancer incidence and mortality from many of the Caribbean islands. In addition, not only do the smaller Caribbean islands lack adequate resources and/or expertise to conduct appropriate epidemiological studies, but many also do not have centralized reporting of new cancer cases. Therefore, while apparent differences in cancer incidence in the Caribbean might be real, they could also be due to inadequate reporting, diagnosis, and/or screening. Due to the limited number of studies conducted in the Caribbean for some cancer-associated viral infections, such as HPV and HHV8 [6], it remains unclear what the true overall prevalences of these viruses are in this population. Therefore, there is a need for a nation-wide Caribbean investigation of cancer-associated viral prevalence. These data, along with reliable data on cancer incidence, would improve current knowledge of regional prevalence and would contribute to the development of cancer prevention strategies.

## Future directions

The AC^3 ^meeting participants define the following areas as priorities for the study of cancer in the Caribbean islands: the need for a Cancer Control program to be written into the Health Plans of each island in order to improve the current knowledge of regional prevalence and to develop cancer prevention strategies; and the need for reliable nation-wide cancer registration in order to provide standardized data on cancer incidence and mortality throughout the Caribbean islands.

The overall purpose of this meeting was to introduce the concept of an African-Caribbean Cancer Consortium and it was well received. The participants felt that the establishment of a Consortium focused on studies of individuals of African descent is warranted. Future AC^3 ^goals were presented by Dr. Camille Ragin and were as follows: 1) the AC^3 ^will provide a forum for the formalization and coordination of collaborations between the investigators from the Unites States, Caribbean, and Africa 2) the AC^3 ^meetings will serve as a medium for these investigators to present their data and to formally discuss the coordination of future collaborations and to seek funding to support case-control studies of cancer risk across these populations. The next AC^3 ^meeting is currently in the planning stages. All of the participants who attended this 2007 meeting agreed that the definition of a clear plan for the AC^3 ^would be the next step that will be addressed during the next meeting.

## Competing interests

The authors of this paper have not received reimbursements, fees, funding, or salary from an organization that may in any way gain or lose financially from the publication of this manuscript, either now or in the future. The authors do not hold any stocks or shares in an organization that may in any way gain or lose financially from the publication of this manuscript, either now or in the future. The authors do not hold or are currently applying for any patents relating to the content of the manuscript nor have received reimbursements, fees, funding, or salary from an organization that holds or has applied for patents relating to the content of the manuscript. The authors have no other financial competing interests or non-financial competing interests (political, personal, religious, ideological, academic, intellectual, commercial or any other) to declare in relation to this manuscript.

## Authors' contributions

CR wrote the manuscript.

CR and CB facilitated the meeting.

CR, CB, AP, AH, NA and MJ made oral presentations during the meeting.

All of the authors read, contributed edits, and approved the final draft of the manuscript.

## References

[B1] Phillips AA, Jacobson JS, Magai C, Condsedine N, Mehler N, Neugut AI (2007). Cancer Incidence and Mortality in the Caribbean. Cancer Investigation.

[B2] Bosch FX, Lorincz A, Munoz N, Meijer CJLM, Shah KV (2002). The causal relation between human papillomavirus and cervical cancer. Journal of Clinical Pathology.

[B3] Hino O, Kajino K (1994). Hepatitis virus-related hepatocarcinogenesis. Intervirology.

[B4] Astori G, Merluzzi S, Arzese A, Brosolo P, de Pretis G, Maieron R, Pipan C, Botta GA (2001). Detection of human papillomavirus DNA and p53 gene mutations in esophageal cancer samples and adjacent normal mucosa. Digestion.

[B5] Shi Y, Li J, Li M, Wang L, Guo W, Qin D, Geng C, Forman D, Newell DG, Peto R, Blot WJ (1997). Association of Helicobacter pylori infection with precancerous lesions and stomach cancer: a case-control study in Yangzhong County. Chin Med Sci J.

[B6] Ragin CC, Kuo J, Taioli E (2007). Prevalence of cancer-associated viruses in the Caribbean islands. Cancer Investigation.

[B7] Ferlay J, Bray F, Pisani P, Parkin DM (2004). GLOBOCAN 2002 Cancer Incidence, Mortality and Prevalence Worldwide IARC CancerBase,Version 2.0 No. 5.

[B8] Watt A, Ragin C, Younger N, Garwood D, Jackson M, Smikle M, Fletcher H, McFarlane-Anderson N (2007). High-risk human papillomavirus (HPV) genotypes in Jamaican women [abstract]. AACR Meeting Abstracts, Apr 2007.

[B9] Fletcher H (1999). Screening for cervical cancer in Jamaica. Caribb Health.

[B10] Clifford GM, Smith JS, Plummer M, Munoz N, Franceschi S (2003). Human papillomavirus types in invasive cervical cancer worldwide: a meta-analysis. Br J Cancer.

[B11] Bunker CH, Zmuda JM, Patrick AL, Wheeler VW, Weissfeld JL, Kuller LH, Cauley JA (2006). High bone density is associated with prostate cancer in older Afro-Caribbean men: Tobago prostate survey. Cancer Causes Control.

[B12] Bunker CH, Patrick AL, Miljkovic-Gacic I, Konety BR, Belle A, Richard JR, Dhir R (2004). Prostate cancer screening parameters in a high-risk African-Caribbean population. Urology.

[B13] Hoffman LJ, Bunker CH, Pellett PE, Trump DL, Patrick AL, Dollard SC, Keenan HA, Jenkins FJ (2004). Elevated seroprevalence of human herpesvirus 8 among men with prostate cancer. J Infect Dis.

[B14] Bunker CH, Patrick AL, Maharaj G, Keenan HA, Ramnarine S, Belle A, Richard JR, Dhir R (2002). Prostate cancer risk is three-fold higher among men, aged 50-64, of African descent compared with men of Asian-Indian descent in Trinidad and Tobago. Ethn Dis.

[B15] Shea PR, Ferrell RE, Patrick AL, Kuller LH, Bunker CH (2002). ELAC2 and prostate cancer risk in Afro-Caribbeans of Tobago. Hum Genet.

[B16] Bunker CH, Patrick AL, Konety BR, Dhir R, Brufsky AM, Vivas CA, Becich MJ, Trump DL, Kuller LH (2002). High prevalence of screening-detected prostate cancer among Afro-Caribbeans: the Tobago Prostate Cancer Survey. Cancer Epidemiol Biomarkers Prev.

[B17] Dieye M, Veronique-Baudin J, Draganescu C, Azaloux H (2007). Cancer incidence in Martinique: a model of epidemiological transition.. European Journal of Cancer Prevention.

[B18] Martin AA, Galan YH, Rodriguez AJ, Graupera M, Lorenzo-Luaces P, Fernandez LM, Camacho R, Lezcano M (1998). The Cuban National Cancer Registry: 1986-1990. Eur J Epidemiol.

[B19] Camacho R, Fernández LM, Martín AA, Abascal ME, Diez M (1994). El programa nacional de control de cáncer en Cuba. Rev Cub Med Gen Integral.

[B20] Freni SC, Freni-Titulaer LW (1981). Cancer incidence in the Netherlands Antilles: a survey covering the period 1968--1979. Cancer.

[B21] (2006). Guyana Cancer Registry, Guyana Cancer Report, 2000-2004. http://www.health.gov.gy/documents.html.

[B22] (2006). U.S.Census Bureau,Population Division/International Programs Center. http://www.census.gov/ipc/www/idb/.

[B23] National Institutes of Health, National Center for Research Resources. http://www.ncrr.nih.gov/.

[B24] NIH Roadmap for Medical Research, Re-engineering the Clinical Research Enterprise. http://nihroadmap.nih.gov/clinicalresearch/overview-translational.asp.

